# Tumor-associated lymphatic vessel density is a reliable biomarker for prognosis of esophageal cancer after radical resection: a systemic review and meta-analysis

**DOI:** 10.3389/fimmu.2024.1453482

**Published:** 2024-09-20

**Authors:** Jin Li, Qing-Bo Wang, Yu-Bo Liang, Xing-Ming Chen, Wan-Ling Luo, Yu-Kai Li, Xiong Chen, Qi-Yu Lu, Yang Ke

**Affiliations:** ^1^ Department of Hepatobiliary Surgery, The Second Affiliated Hospital, Kunming Medical University, Kunming, China; ^2^ Department of Oncology, Mengchao Hepatobiliary Hospital of Fujian Medical University, Fuzhou, China; ^3^ Department of Gastrointestinal Surgery, The Second Affiliated Hospital, Kunming Medical University, Kunming, China; ^4^ Department of Surgical Education and Research, The Second Affiliated Hospital, Kunming Medical University, Kunming, China; ^5^ Yunnan Yunke Bio-Technology Institute, Kunming, China

**Keywords:** esophageal cancer, lymphangiogenesis, lymphatic vessel density, meta-analysis, overall survival, prognosis, radical resection, recurrence-free survival

## Abstract

**Purpose:**

To explore whether tumor-associated lymphatic vessel density (LVD) could be a biomarker for the prognosis of patients with esophageal cancer after radical resection.

**Methods:**

A systematic literature search was performed through PubMed, EMBASE, Wanfang Data, and Cochrane Library from the inception of databases until March 19, 2024. The selected studies investigated overall survival (OS) and/or recurrence-free survival (RFS) of patients with esophageal cancer with different levels of LVD after radical resection. The OS and RFS data were pooled as hazard ratios (HR) and 95% confidential interval (CI). Furthermore, the standardized mean differences (SMDs) and 95% CI were aggregated to evaluate the correlation between LVD and clinicopathological features.

**Results:**

A total of 10 retrospective studies of 1,201 patients were finally included for the meta-analysis. Patients with esophageal cancer with a high level of LVD exhibited worse OS (HR 1.65, 95% CI 1.18 to 2.31) and RFS (HR 1.57, 95% CI 1.09 to 2.26) than those with a low level of LVD. Subgroup analysis of different pathological subtypes revealed that patients with esophageal adenocarcinoma with a high level of LVD had significantly worse RFS (HR 2.84, 95% CI 1.61 to 5.02) than those with a low level of LVD; while patients with esophageal squamous cell carcinoma with a high level of LVD had similar OS (HR 1.52, 95% CI 0.93 to 2.47) and RFS (HR 1.03, 95% CI 0.72 to 1.48) to those with a low level of LVD. Furthermore, tumors with lymph node metastasis had significantly higher levels of LVD than those without lymph node metastasis (SMD = 1.11, 95% CI 0.54 to 1.67). Tumors at the stages III-IV had significantly higher levels of LVD than those at the stages I-II (SMD = 1.62, 95% CI 0.90 to 2.34).

**Conclusion:**

A high level of LVD in tumor was associated with worse survival of patients with esophageal cancer after radical resection, especially in patients with esophageal adenocarcinoma. Tumor-associated LVD is a new parameter that should be measured in postoperative pathology for predicting the prognosis of patients with esophageal cancer.

**Systematic review registration:**

https://www.crd.york.ac.uk/prospero/ PROSPERO, identifier CRD42024553766.

## Introduction

Esophageal cancer (EC) is one of the frequently diagnosed cancers and leading causes of cancer-related deaths worldwide, with an estimated 511,000 new cases and 445,000 deaths in the world, according to global cancer statistics in 2022 (1). Pathologically, EC is classified into esophageal adenocarcinoma (EAC) and esophageal squamous cell carcinoma (ESCC) as well as other rare types (2). EAC typically occurs in the lower third of the esophagus and is more common in developed countries, while ESCC typically occurs in the upper two-thirds of the esophagus and is more common in developing countries (3–5). Although radical resection is the most optimal therapeutic approach for early-stage EC, the prognosis remains dismal with a 5-year overall survival (OS) rate of approximately 13%-18% (6–8). It is crucial to identify novel prognostic markers to manage EC patients (9).

Tumor-associated lymphangiogenesis is the formation of new lymphatic vessels in tumor, which is driven by growth factors secreted from tumor cells and cells in the tumor microenvironment (10, 11). The lymphangiogenesis can be quantified by measuring lymphatic vessel density (LVD) (12, 13). High levels of LVD in tumor are associated with the worse survival of patients with hepatocellular carcinoma (13), intrahepatic cholangiocarcinoma (14), and hilar cholangiocarcinoma (15) after radical resection. Previous studies have reported that the levels of LVD are associated with worse survival of patients with EC after radical resection (16–23), while others have shown that the levels of LVD are not significantly associated with the prognosis of patients with EC after radical resection (24, 25). The difference may stem from a relatively smaller sample size (16–25). Hence, the prognostic value of LVD for EC is not clarified.

In this study, we performed a systemic review and meta-analysis to clarify the role of lymphangiogenesis in the prognosis of EC, and whether LVD could be a prognostic indicator for patients with EC after radical resection.

## Materials and methods

This systematic review and meta-analysis were conducted following the Preferred Reporting Items for Systematic Reviews and Meta-Analyses (PRISMA) statement (26), and the study protocol was registered in an official repository (PROSPERO registration number: CRD42024553766).

### Search strategy

A systematic literature search of PubMed, EMBASE, Wanfang Data, and Cochrane Library was performed, from the inception of databases to March 19, 2024, to identify all relevant studies on the association between LVD and survival outcomes in patients with EC after radical resection. The search strategy utilized the terms: (“lymphangiogenesis” OR “lymphangiogeneses” OR “lymph vessel” OR “lymphatic vessel” OR “lymph microvessel” OR “lymphatic microvessel” OR “podoplanin” OR “D2-40”) AND (“esophageal” OR “oesophageal”). Filters for study types, geographical location, or language limits were not applied. The reference lists of the retrieved articles were also consulted.

### Article selection

Two investigators (J.L. and Q.B.W.) independently reviewed the titles, abstracts, and full texts of the articles, based on the pre-specified inclusion criteria: (1) studies focusing on EC patients, who underwent the radical resection; (2) determining LVD in tumor samples using immunohistochemistry; (3) categorizing patients into high and low LVD groups, based on a specified cut-off value; (4) reporting survival outcomes of OS and/or recurrence-free survival (RFS). Studies were excluded based on the following criteria: (1) duplication, only the study with the largest sample size was included if the same population was used in multiple studies; (2) non-clinical study articles: e.g., case report, conference abstract, review, meta-analysis.

### Data extraction

The relevant information was extracted from every eligible article by two independent reviewers (J.L. and Q.B.W.) in predefined formats, including the following items: the first author’s name, year of publication, country/region, the sample size of groups with different levels of LVD, cut-off value of LVD, sex, mean/median age, the pathological subtypes of the tumor, differentiation grade, location of tumor, the extent of the tumor invasion, lymph node invasive status, TNM stage, median follow up, OS, RFS, hazard ratio (HR), and corresponding 95% confidence interval (CI). The mean and standard deviation (SD) of LVD in groups with or without lymph node metastasis and groups with different TNM stages were also extracted. Unavailable raw data from articles were tried to obtain from the corresponding authors. If HR and 95% CI were not available, they were calculated from Kaplan-Meier curves using Tierney’s method (27).

### Quality assessment

The quality of the included studies was assessed by two independent reviewers (J.L. and Q.B.W.) using the Newcastle-Ottawa scale (NOS). Studies with scores between 7 and 9 were considered good quality, studies with scores between 4 and 6 were considered fair quality, while studies with a score of ≤ 3 were considered low quality (28, 29). In case of discrepancies between the investigators (J.L. and Q.B.W.) during the processes of article selection, data extraction, and quality assessment, a senior reviewer (X.C. or Q.Y.L. or Y.K.) participated in a discussion to make a final decision.

### Statistical analyses

The HR and 95% CI were aggregated to evaluate the effect of LVD on OS and RFS in patients with EC after radical resection. A pooled HR greater than 1 and a 95% CI that did not cross 1 indicated a worse survival outcome in the group with a high level of LVD compared to that with a low level of LVD. The standardized mean difference (SMD) and 95% CI were aggregated to evaluate the correlation between the levels of LVD and lymph node metastasis or higher TNM stages. A pooled SMD greater than 0 and a 95% CI that did not cross 0 indicated a higher level of LVD in groups with lymph node metastasis or higher TNM stages. The I-square (I^2^) test was utilized to assess the heterogeneity of the meta-analysis results, with a threshold of 50% considered significant heterogeneity (30). In cases where I^2^ exceeded 50%, the random effects model was chosen to calculate the combined estimates, otherwise, the fixed effects model was applied (31, 32). Subgroup analysis was performed based on the pathological subtypes of EC. Egger’s and Begg’s regression analysis of funnel plot was conducted to evaluate any possible publication bias (33). All the statistical analyses were conducted by the Stata 18.0 software (Stata, Texas, USA).

## Results

### Study selection

**Figure 1:**
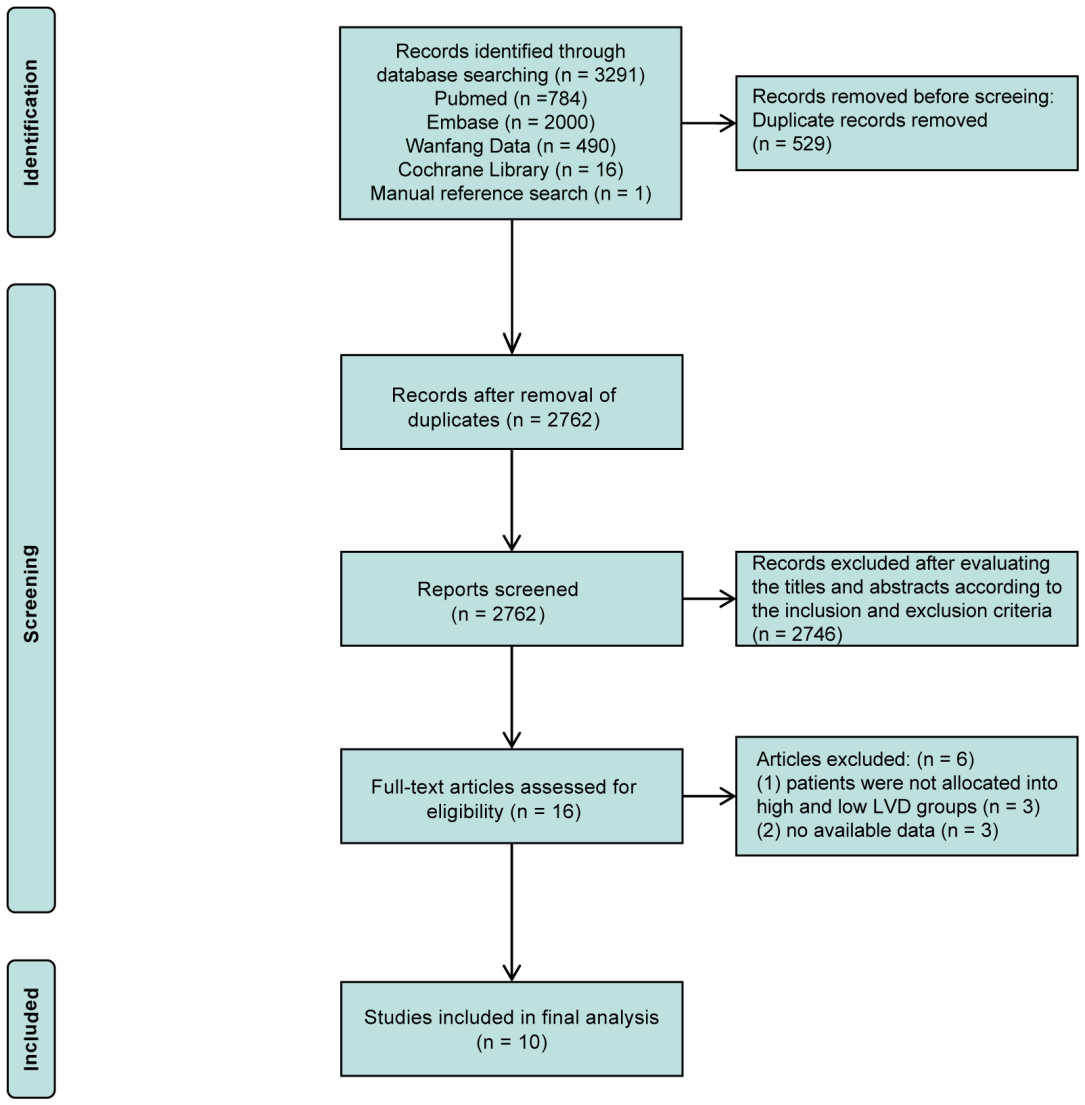
PRISMA flow diagram of the literature search and selection.

The comprehensive literature search process is illustrated in the flow diagram (**Figure 1**). A total of 3,291 records were retrieved from PubMed (784), EMBASE (2,000), Wanfang Data (490), Cochrane Library (16), and reference lists (1). After removing duplications, 2,762 records remained, and of them, 16 studies underwent full-text evaluation after the exclusion of 2,741 records through evaluating their titles and abstracts, according to the inclusion and exclusion criteria. Subsequently, 6 studies were excluded for reasons, such as the absence of subgroups into high and low LVD groups and no available data. Ultimately, 10 retrospective studies were included in the meta-analysis (16–25), and most studies had high quality, except one with fair quality (**Supplementary Table 1**).

### Study and patient characteristics

The baseline characteristics of the 10 eligible studies are presented in **Table 1**. The studies covered 14 years (2007-2021). Seven studies were conducted in China (16–19, 21, 23, 24), one in Japan (25), one in Austria (22), and one in Poland (20). A total of 1,201 patients with EC after radical resection were included in these studies, with 927 male (77%) and 274 female (23%), and an age ranged from 35 to 81 years old. The degrees of LVD were evaluated by immunohistochemistry with the lymphatic endothelial cell marker D2-40 (a.k.a. podoplanin) in all 10 studies. Patients were stratified into the high and low LVD groups, based on median LVD in five studies (18, 20–22, 24), mean LVD in one study (25), and a specific cut-off value in the remaining four studies (16, 17, 19, 23). Among the 10 studies, 6 studies reported the number of patients with different levels of LVD in tumor (17–19, 22–24), including 454 (48.8%) patients with a high level of LVD in tumor and 477 (51.2%) patients with a low level of LVD in tumor, while other 4 studies did not reported the number of patients with different levels of LVD in tumor, but reported HR and 95% CI about the role of different levels of LVD in tumor on prognosis (16, 20, 21, 25). Baseline comparisons between the group with a high level of LVD and the group with a low level of LVD were performed in 9 studies (17–25). There was no significant difference in sex, age, pathologic subtypes, differentiation grade, and tumor location between these two groups. The median follow-up time periods ranged from 28.5 to 54.4 months.

**Table 1:** Characteristics of patients with esophageal cancer with high and low levels of lymphatic vessel density after radical resection.

Ref.	Year of publication	Country	No. of patient (high vs. low)	Cut-off value	No. of Male (%)	Mean or median age (yr)	Pathologic subtypes EAC (n)/ ESCC (n)	Location of tumor (upper/midthoracic/lower)	Differentiation grade (good/moderate/poor)	Extent of tumor invasion (T1/T2/T3/T4)	Lymph node invasion (Y/N)	TNM (I/II/III/IV)	Median follow-up (M)
Nakayama Y et al. (25)	2007	Japan	29^¶^	7.2/HPF	23 (79.3)	61.3	EAC (21)/ ESCC (8)	NA	3/20/5	4/5/16/4	9/20	3/6/12/8^※^	54.4
Li CH et al. (24)	2008	China	54 vs. 24	7.4/HPF	56 (71.8)	53.8	ESCC (78)	NA	36/30/11	3/12/60/3	55/23	2/49/24/3^*^	33.0
Bu XH et al. (16)	2012	China	60^¶^	25/HPF	40 (66.7)	58.0	ESCC (60)	NA	good/moderate + poor: 20/40	NA	34/26	NA^※^	49.5
Schoppmann SF et al. (22)	2013	Austria	164 vs. 181	12/HPF	269 (77.9)	63.0	EAC (194)/ ESCC (151)	NA	17/181/147	58/94/177/16	122/203	58/94/177/16^⁑^	52.0
Kozlowski M et al. (20)	2013	Poland	74^¶^	38/HPF	60 (81.1)	66.0	EAC (42)/ ESCC (32)	4/26/44	7/32/35	T1+T2/T3+T4: 21/53	25/49	NA^⁑^	28.5
Xie LX et al. (23)	2013	China	61 vs. 67	13/HPF	106 (82.8)	60.0	EAC (128)	NA	good + moderate/poor: 51/77	T1/T2+T3/T4: 0/85/43	42/86	NA^*^	45.0
Ma W et al. (21)	2014	China	107^¶^	4.4/HPF	85 (79.4)	60.0	ESCC (107)	11/66/36	25/53/29	14/23/62/8	69/38	17/53/37/0^⁑^	40.0
Chen B et al. (18)	2014	China	54 vs. 70	median (?)	98 (79.0)	59.0	ESCC (124)	NA	22/93/9	T1+T2/T3+T4: 17/107	58/66	I+II/III+IV: 66/58^⁑^	NA
Chen GQ et al. (19)	2014	China	83 vs. 123	17/HPF	152 (73.7)	58.6	ESCC (206)	25/113/68	40/118/48	33/110/63/0	NA	NA^⁑^	55.9
Tang SJ et al. (17)	2021	China	38 vs. 12	3/HPF	38 (76.0)	62.5	ESCC (50)	upper + midthoracic/lower: 30/20	12/18/20	T1+T2/T3+T4: 15/35	26/24	NA^⁂^	NA

¶, the total number of high and low groups; *, adopted to the 6^th^ edition of AJCC/UICC TNM staging for esophageal cancer; ⁑, adopted to the 7^th^ edition of AJCC/UICC TNM staging for esophageal cancer; ⁂, adopted to the 8^th^ edition of AJCC/UICC TNM staging for esophageal cancer; ※, not available of version information of TNM staging; EAC, esophageal adenocarcinoma; ESCC, esophageal squamous cell carcinoma; HPF, high-power field; NA, not available; TNM, tumor node metastasis classification.

### Overall survival

**Figure 2:**
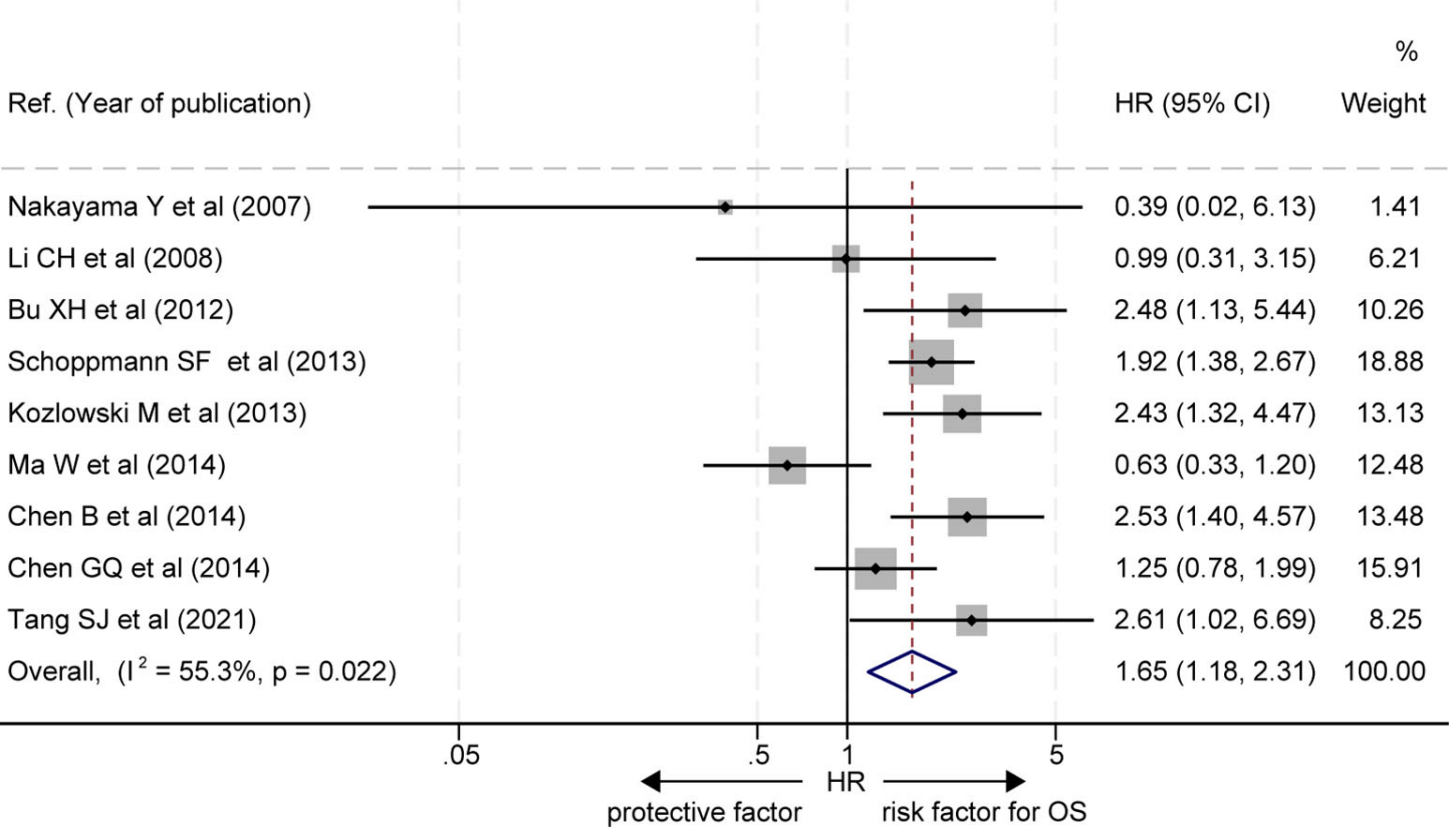
Comparison of overall survival between patients with esophageal cancer with a high level of LVD and those with a low level of LVD after radical resection. CI, confidence interval; HR, hazard ratio; LVD, lymphatic vessel density.

The effects of LVD on OS were assessed in 9 studies (16–22, 24, 25) (**Table 2**). Seven studies reported that patients with a high level of LVD in tumor had significantly worse OS than those with a low level of LVD in tumor (16–22), and two studies reported that patients with a high level of LVD in tumor had a similar OS to those with a low level of LVD in tumor (24, 25). Data pooled from those 9 studies exhibited that patients with a high level of LVD in tumor had significantly worse OS than those with a low level of LVD in tumor (HR = 1.65, 95% CI 1.18 to 2.31, **Figure 2**).

**Table 2:** Overall survival of patients with esophageal cancer with high and low levels of lymphatic vessel density after radical resection.

Ref.	Year of publication	Group	Overall survival			
			One-year	Three-year	Five-year	P (log-rank)
Nakayama Y et al. (25)	2007	High	73.44%	34.36%	29.40%	0.083
		Low	89.72%	64.77%	62.50%	
Li CH et al. (24)	2008	High	93.99%	42.30%	NA	0.283
		Low	96.63%	52.20%	NA	
Bu XH et al. (16)	2012	High	77.00%	46.50%	38.90%	< 0.050
		Low	98.70%	84.51%	62.50%	
Schoppmann SF et al. (22)	2013	High	80.00%	36.00%	30.00%	< 0.001
		Low	85.00%	61.00%	54.00%	
Kozlowski M et al. (20)	2013	High	74.98%	26.17%	19.03%	0.003
		Low	97.56%	62.16%	49.00%	
Ma W et al. (21)	2014	High	81.38%	43.90%	NA	0.042
		Low	92.78%	59.10%	NA	
Chen B et al. (18)	2014	High	83.05%	24.10%	12.40%	< 0.001
		Low	88.76%	65.79%	56.57%	
Chen GQ et al. (19)	2014	High	93.14%	80.96%	54.20%	0.017
		Low	94.19%	84.61%	71.50%	
Tang SJ et al. (17)	2021	High	71.10%	34.20%	21.10%	< 0.050
		Low	83.30%	66.70%	58.30%	

**Figure 3:**
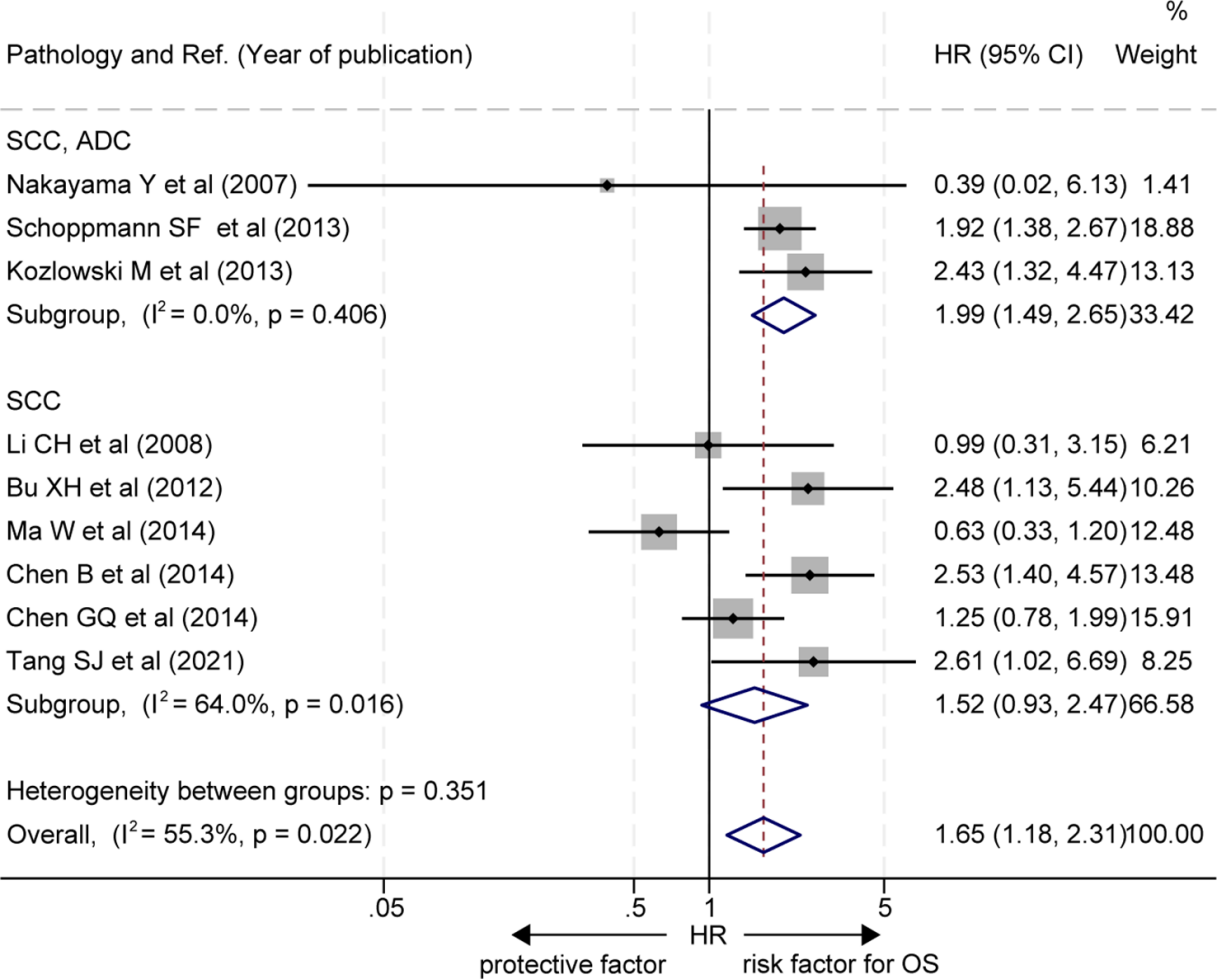
Subgroup comparison of overall survival between patients with esophageal cancer with a high level of LVD and those with a low level of LVD based on pathologic subtypes. CI, confidence interval; EAC, esophageal adenocarcinoma; ESCC, esophageal squamous cell carcinoma; HR, hazard ratio; LVD, lymphatic vessel density.

In subgroup analysis based on pathological subtypes of EC, three out of the 9 studies did not stratify for EAC or ESCC subgroups (20, 22, 25), and the OS of patients with a high level of LVD in tumor was significantly worse than those with a low level of LVD in these studies (HR = 1.99, 95% CI 1.49 to 2.65, **Figure 3**, the upper panel). Six studies focused on the ESCC subgroup (16–19, 21, 24), and patients with a high level of LVD in tumor had OS, similar to those with a low level of LVD in these studies (HR = 1.52, 95% CI 0.93 to 2.47, **Figure 3**, the lower panel).

### Recurrence-free survival

**Figure 4:**
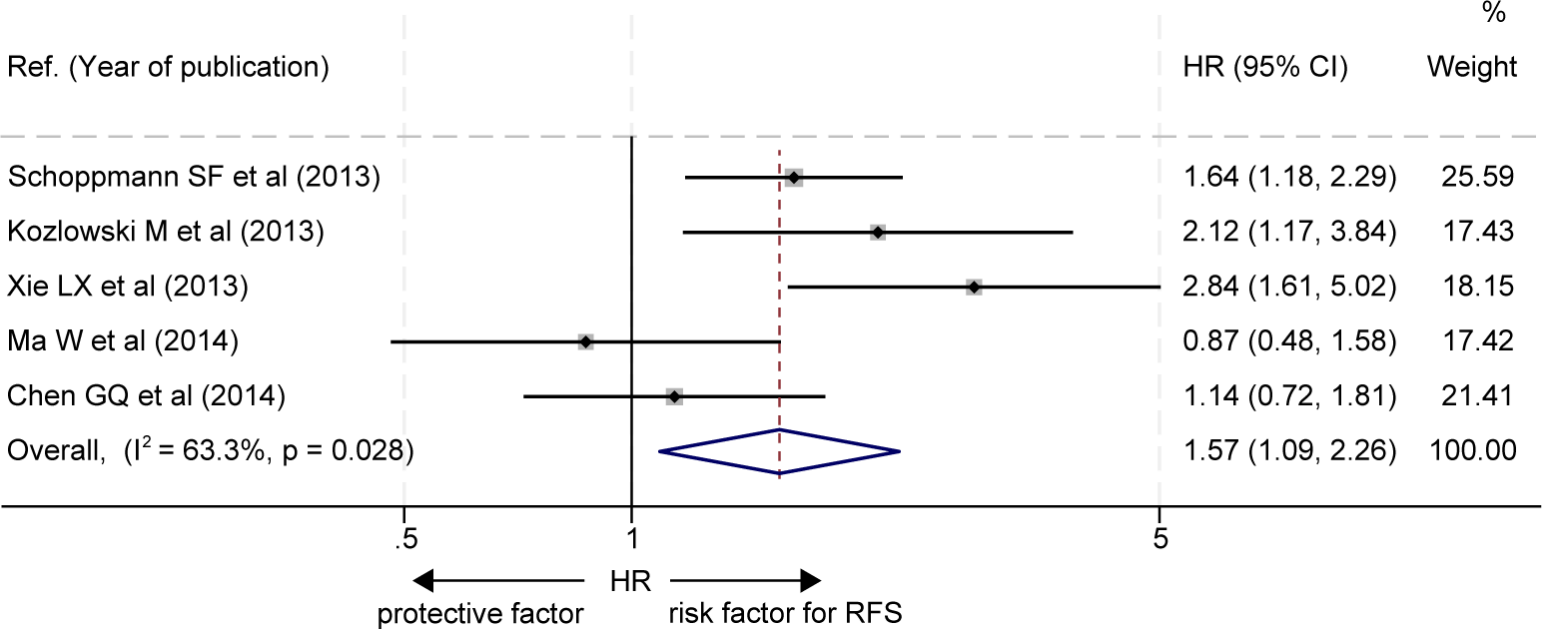
Comparison of recurrence-free survival between patients with esophageal cancer with a high level of LVD and those with a low level of LVD after radical resection. CI, confidence interval; HR, hazard ratio; LVD, lymphatic vessel density.

The effects of LVD on RFS were assessed in 5 studies (19–23) (**Table 3**). Five studies consistently reported that patients with a high level of LVD in tumor had significantly worse RFS than those with a low level of LVD in tumor (19–23). Data pooled from those 5 studies indicated that patients with a high level of LVD in tumor had significantly worse RFS than those with a low level of LVD in tumor (HR = 1.57, 95% CI 1.09 to 2.26, **Figure 4**).

**Table 3:** Recurrence-free survival of patients with esophageal cancer with high and low levels of lymphatic vessel density after radical resection.

Ref.	Year of publication	Group	Recurrence-free survival			
			One-year	Three-year	Five-year	P (log-rank)
Schoppmann SF et al. (22)	2013	High	62.00%	32.00%	28.00%	< 0.001
		Low	75.00%	49.00%	48.00%	
Kozlowski M et al. (20)	2013	High	62.20%	23.76%	15.87%	0.010
		Low	86.44%	52.03%	31.74%	
Xie LX et al. (23)	2013	High	72.31%	21.32%	12.48%	< 0.001
		Low	88.15%	60.56%	53.22%	
Ma W et al. (21)	2014	High	64.44%	43.90%	NA	0.035
		Low	90.35%	60.60%	NA	
Chen GQ et al. (19)	2014	High	88.74%	74.10%	56.48%	0.030
		Low	91.55%	80.10%	71.27%	

**Figure 5:**
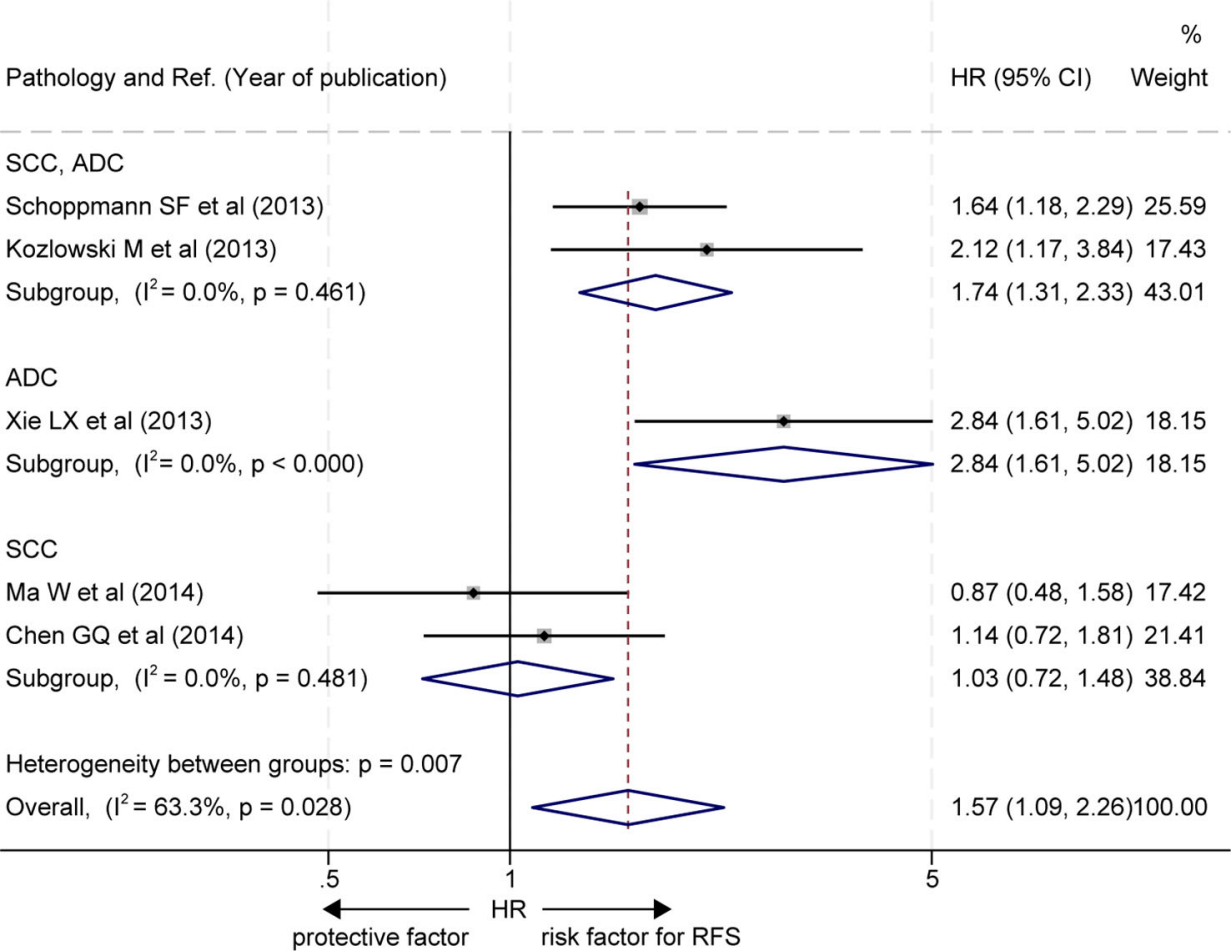
Subgroup comparison of recurrence-free survival between patients with esophageal cancer with a high level of LVD and those with a low level of LVD based on pathologic subtypes. CI, confidence interval; EAC, esophageal adenocarcinoma; ESCC, esophageal squamous cell carcinoma; HR, hazard ratio; LVD, lymphatic vessel density.

Furthermore, two out of the five studies did not stratify EC pathological subgroups (20, 22), and the RFS of patients with a high level of LVD in tumor was significantly worse than those with a low level of LVD in these studies (HR = 1.74, 95% CI 1.31 to 2.33, **Figure 5**, the upper panel). One study focused on the EAC subgroup (23), and the RFS of patients with a high level of LVD in tumor was significantly worse than those with a low level of LVD in the study (HR = 2.84, 95% CI 1.61 to 5.02, **Figure 5**, the middle panel). Two studies focused on the ESCC subgroup (19, 21), and patients with a high level of LVD in tumor had RFS, similar to those with a low level of LVD in these studies (HR = 1.03, 95% CI 0.72 to 1.48, **Figure 5**, the lower panel).

### Correlation between the levels of LVD and lymph node metastasis

**Figure 6:**
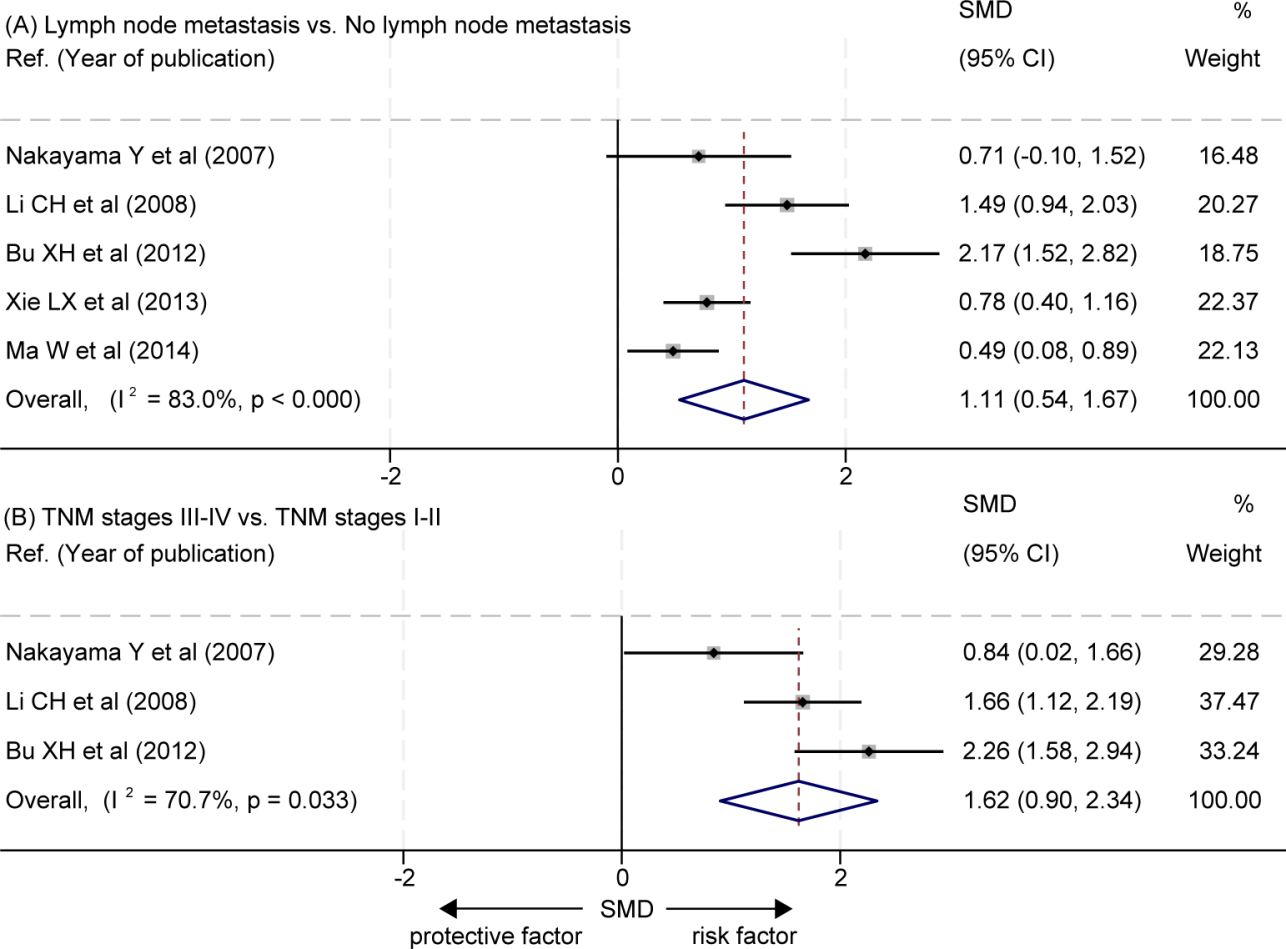
Correlation between the levels of LVD and clinicopathological features in patients with esophageal cancer after radical resection. **(A)** Correlation between the levels of LVD and lymph node metastasis. **(B)** Correlation between the levels of LVD and TNM stages. CI, confidence interval; LVD, lymphatic vessel density; SMD, standardized mean difference; TNM, tumor node metastasis classification.

The data about the correlation between the levels of LVD and lymph node metastasis was extracted from five studies (16, 21, 23–25) (**Table 4**). Four studies reported that tumors with lymph node metastasis had significantly higher levels of LVD than those without lymph node metastasis (16, 21, 23, 24), and one study reported that tumors with lymph node metastasis had a similar level of LVD to those without lymph node metastasis (25). Data pooled from those five studies indicated that tumors with lymph node metastasis had significantly higher levels of LVD than those without lymph node metastasis (SMD = 1.11, 95% CI 0.54 to 1.67, **Figure 6A**).

**Table 4:** Correlation between the levels of lymphatic vessel density and lymph node metastasis in patients with esophageal cancer after radical resection.

Ref.	Year of publication	Lymph node metastasis	No.	Lymphatic vessel density		P
				Mean	SD	
Nakayama Y et al. (25)	2007	Positive	20	7.97	3.26	0.0745
		Negative	9	5.60	3.21	
Li CH et al. (24)	2008	Positive	23	7.73	1.86	0.030
		Negative	55	5.38	1.43	
Bu XH et al. (16)	2012	Positive	26	37.79	4.99	0.001
		Negative	34	25.82	5.76	
Xie LX et al. (23)	2013	Positive	86	16.651	6.073	0.001
		Negative	42	12.319	4.096	
Ma W et al. (21)	2014	Positive	38	6.09	4.06	0.010
		Negative	69	4.15	3.92	

SD, standard deviation.

### Correlation between the levels of LVD and higher TNM stages

The data about the correlation between the levels of LVD and TNM stages was extracted from three studies (16, 24, 25) (**Table 5**). The three studies consistently reported that tumors at the stages III-IV had significantly higher levels of LVD than those at the stages I-II (16, 24, 25). The pooled data also indicated that tumors at the stages III-IV had significantly higher levels of LVD than those at the stages I-II (SMD = 1.62, 95% CI 0.90 to 2.34, **Figure 6B**).

**Table 5:** Correlation between the levels of lymphatic vessel density and TNM stages in patients with esophageal cancer after radical resection.

Ref.	Year of publication	TNM stages	No.	Lymphatic vessel density		P
				Mean	SD	
Nakayama Y et al. (25)	2007	III-IV	20	8.16	3.15	0.0351
		I-II	9	5.47	3.00	
Li CH et al. (24)	2008	III-IV	27	7.18	1.35	0.013
		I-II	51	4.27	1.91	
Bu XH et al. (16)	2012	III-IV	20	39.37	3.52	0.001
		I-II	40	26.83	6.20	

SD, standard deviation; TNM, tumor node metastasis classification.

### Publication bias

There was no significant publication bias in funnel plots for OS (Egger’s *P* = 0.428 and Begg’s *P* = 0.602, **Supplementary Figure 1A**) or RFS (Egger’s *P* = 0.724 and Begg’s *P* = 0.807, **Supplementary Figure 1B**).

## Discussion

This study investigated the potential role of tumor-associated LVD in the prognosis of patients with EC after radical resection. While earlier studies have explored the impact of different levels of tumor-associated LVD on the prognosis of patients with EC after radical resection (16–25), the findings from these studies remain controversial due to the relatively smaller sample size. Therefore, we conducted this meta-analysis of 1,201 cases from 10 studies and our findings revealed that a high level of LVD in tumor was associated with worse OS and RFS of patients with EC after radical resection. Hence, tumor-associated lymphangiogenesis may have an unfavorable impact on the prognosis of patients with EC. This is a key inference in our study, and some evidence supports the inference: firstly, lymph node metastasis is a well-established prognostic factor for recurrence and poor outcomes in patients with EC after radical resection (34). With the increase in the number of positive lymph nodes, the OS rate of patients with EC decreases significantly (35). Secondly, lymphatic vessels function as a channel for malignant tumor cells to metastasize into lymph nodes and distant organs (36). The step of tumor cell entry into lymphatic vessels was termed lymphatic vascular invasion (37). Patients with EC with positive lymphatic vascular invasion have a worse prognosis compared to those without lymphatic vascular invasion (38–40). Thirdly, a high level of tumor-associated LVD probably increases the probability of lymphatic vascular invasion and lymph node metastasis (41, 42), based on the fact that an increase in LVD correlates with a higher probability of lymph node metastasis and higher TNM stages (17, 18, 20, 23, 24). Given that tumor-associated lymphangiogenesis may be an early and essential event of lymphatic vessel invasion and lymph node metastasis, future studies may explore the value of LVD as a biomarker of recurrence or prognosis of patients with EC after radical resection when no lymphatic vessel invasion and lymph node metastasis is found.

Subgroup analysis based on pathological subtypes of EC exhibited that ESCC patients had similar OS and RFS periods after radical resection regardless of the levels of LVD in the tumors. However, as regards EAC, the predominant pathological subtype in America and the Western world, patients with a high level of LVD in their tumors had worse RFS than those with a low level of LVD after radical resection. Apparently, tumor-associated LVD had different prognosis roles in ESCC and EAC. The significant role of tumor-associated LVD in EAC may reflect a metastatic tendency of adenocarcinoma, which more relies on the lymphatic system than squamous cell carcinoma (43, 44). Future prospective studies with larger sample sizes may verify our observation. We tried to perform subgroup analysis based on other clinicopathological features, like TNM stages, lymph node metastasis, and tumor cell differentiation. However, no study evaluated the role of LVD in the prognosis of these different subgroups of EC.

We recognized our study had limitations. Firstly, all data included in this study were obtained from retrospective cohort studies. Secondly, most patients in the study came from China, which may reflect the most prevalent region of the most prevalent type, ESCC, worldwide and may reduce the generalizability of our findings to other regions. Thirdly, although the cut-off value of high and low levels of LVD was generally determined by the median or mean value of LVD, no uniform cut-off value was used across different studies, which may increase the heterogeneity in the meta-analysis.

In conclusion, this study systematically reviewed the prognostic outcome of patients with EC with different levels of LVD after radical resection. Patients with EC with a high level of LVD had worse OS and RFS than those with a low level of LVD especially in patients with EAC.

## Data Availability

The original contributions presented in the study are included in the article/**Supplementary Material**. Further inquiries can be directed to the corresponding authors.
