# Supplementary material for: Tumor-associated lymphatic vessel density is a reliable biomarker for prognosis of esophageal cancer after radical resection: a systemic review and meta-analysis
Source: Front Immunol. 2024 Sep 20;15:1453482. doi: 10.3389/fimmu.2024.1453482 (PMC11449706; doi:10.3389/fimmu.2024.1453482)
Supplement: Supplementary file 1 [file DataSheet1.docx]

**Supplementary information**

Supplementary Table:

Supplementary Table 1 Newcastle-Ottawa Scale for the assessment of the quality of the included studies

| Study | Selection (4 points) | | | | Comparability (2 points) | Exposure (3 points) | | | Overall (9 points) |
| --- | --- | --- | --- | --- | --- | --- | --- | --- | --- |
|  | Representative of the Exposed Cohort | Selection of Non-Exposed | Ascertainment of Exposure | Outcome Not Present at Start |  | Assessment of Outcome | Adequate Follow-Up Length | Adequacy of Follow-Up |  |
| Nakayama Y et al | * | * | * | * | * | * | * |  | 7/9 |
| Li CH et al | * | * | * | * | * | * |  | * | 7/9 |
| Bu XH et al | * | * | * | * | * | * | * | * | 8/9 |
| Schoppmann SF et al | * | * | * | * |  | * | * |  | 6/9 |
| Kozlowski M et al | * | * | * | * | * | * | * | * | 8/9 |
| Xie LX et al | * | * | * | * | * | * | * | * | 8/9 |
| Ma W et al | * | * | * | * | * | * |  | * | 7/9 |
| Chen B et al | * | * | * | * | * | * | * |  | 7/9 |
| Chen GQ et al | * | * | * | * | * | * | * | * | 8/9 |
| Tang SJ et al | * | * | * | * | * | * | * |  | 7/9 |

Supplementary Figure and Figure Legend:

**
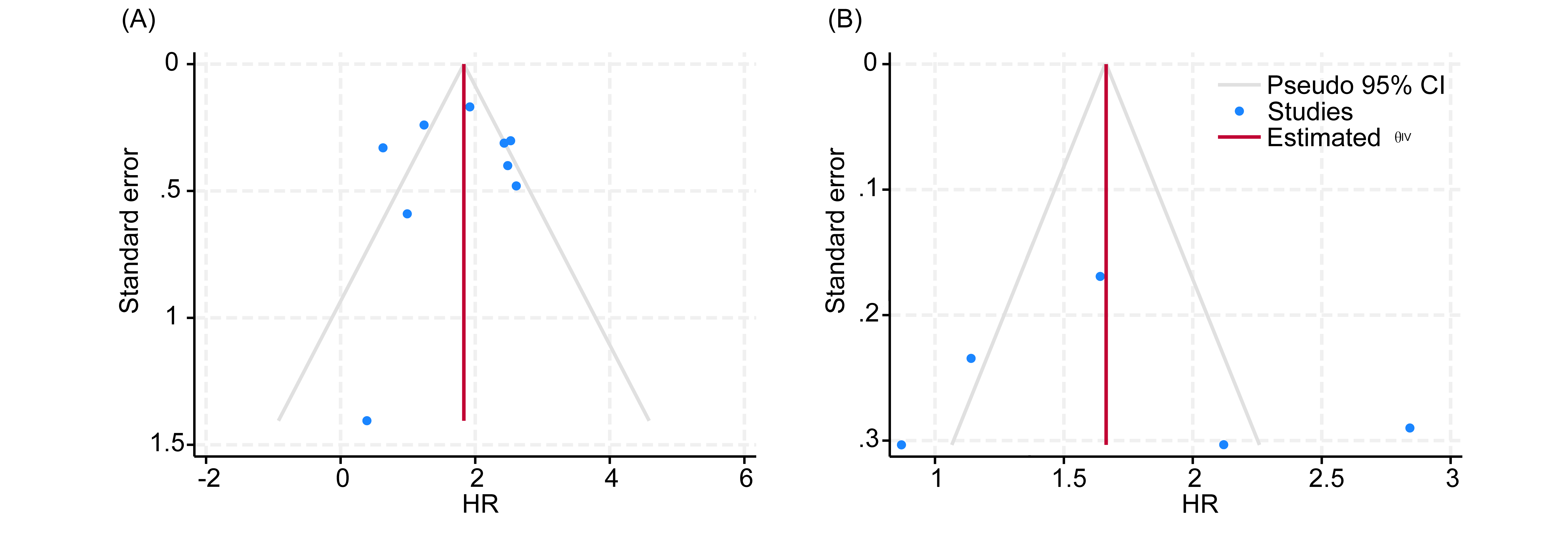
**

Figure S1 Funnel plot for Overall survival (A) and recurrence-free survival (B)
